# A New Influenza-Tracking Smartphone App (Flu-Report) Based on a Self-Administered Questionnaire: Cross-Sectional Study

**DOI:** 10.2196/mhealth.9834

**Published:** 2018-06-06

**Authors:** Kazutoshi Fujibayashi, Hiromizu Takahashi, Mika Tanei, Yuki Uehara, Hirohide Yokokawa, Toshio Naito

**Affiliations:** ^1^ Department of General Medicine School of Medicine Juntendo University Tokyo Japan

**Keywords:** influenza, epidemiology, pandemics, internet, participatory surveillance, participatory epidemiology

## Abstract

**Background:**

Influenza infections can spread rapidly, and influenza outbreaks are a major public health concern worldwide. Early detection of signs of an influenza pandemic is important to prevent global outbreaks. Development of information and communications technologies for influenza surveillance, including participatory surveillance systems involving lay users, has recently increased. Many of these systems can estimate influenza activity faster than the conventional influenza surveillance systems. Unfortunately, few of these influenza-tracking systems are available in Japan.

**Objective:**

This study aimed to evaluate the flu-tracking ability of Flu-Report, a new influenza-tracking mobile phone app that uses a self-administered questionnaire for the early detection of influenza activity.

**Methods:**

Flu-Report was used to collect influenza-related information (ie, dates on which influenza infections were diagnosed) from November 2016 to March 2017. Participants were adult volunteers from throughout Japan, who also provided information about their cohabiting family members. The utility of Flu-Report was evaluated by comparison with the conventional influenza surveillance information and basic information from an existing large-scale influenza-tracking system (an automatic surveillance system based on electronic records of prescription drug purchases).

**Results:**

Information was obtained through Flu-Report for approximately 10,094 volunteers. In total, 2134 participants were aged <20 years, 6958 were aged 20-59 years, and 1002 were aged ≥60 years. Between November 2016 and March 2017, 347 participants reported they had influenza or an influenza-like illness in the 2016 season. Flu-Report-derived influenza infection time series data displayed a good correlation with basic information obtained from the existing influenza surveillance system (rho, ρ=.65, *P*=.001). However, the influenza morbidity ratio for our participants was approximately 25% of the mean influenza morbidity ratio for the Japanese population. The Flu-Report influenza morbidity ratio was 5.06% (108/2134) among those aged <20 years, 3.16% (220/6958) among those aged 20-59 years, and 0.59% (6/1002) among those aged ≥60 years. In contrast, influenza morbidity ratios for Japanese individuals aged <20 years, 20-59 years, and ≥60 years were recently estimated at 31.97% to 37.90%, 8.16% to 9.07%, and 2.71% to 4.39%, respectively.

**Conclusions:**

Flu-Report supports easy access to near real-time information about influenza activity via the accumulation of self-administered questionnaires. However, Flu-Report users may be influenced by selection bias, which is a common issue associated with surveillance using information and communications technologies. Despite this, Flu-Report has the potential to provide basic data that could help detect influenza outbreaks.

## Introduction

Seasonal influenza outbreaks cause 250,000-500,000 deaths worldwide annually and are a major public health concern [1]. Because influenza infections can spread rapidly, early detection of signs of an influenza pandemic is important to prevent global outbreaks. In Japan, the official sentinel surveillance system for influenza takes 1-2 weeks to report data on the intensity of influenza activity [2]. Globally, development of information and communications technologies for influenza surveillance has recently increased [3-5]. These systems can estimate influenza activity faster than the conventional influenza surveillance systems. Unfortunately, few influenza-tracking systems using information and communications technologies are available in Japan. However, a large number of Japanese households are connected to the internet, and 71.8% of households have a mobile phone [6]. Therefore, a mobile phone app focused on collecting influenza-related information may be able to monitor influenza activity more easily and faster than the conventional surveillance systems. “Flu-Report,” a new flu-tracking iPhone app, was developed by the Department of General Medicine, School of Medicine, Juntendo University, and launched in November 2016. Flu-Report collects influenza-related information directly from users based on a self-administered questionnaire. This study aimed to evaluate the flu-tracking ability of Flu-Report.

## Methods

### Ethics Statement

All volunteers who wanted to join the study downloaded Flu-Report (for free) from the iPhone App Store, read the written informed consent document included in the app, and checked the “Agree” button on the consent form. Participants were able to withdraw from the study at any time. The study protocol was approved by the Ethical Review Board of Juntendo University (#2017007).

### Flu-Report

Flu-Report, a new flu-tracking iPhone app, was developed with “ResearchKit,” an open source framework (Apple, One Apple Park Way, Cupertino, CA, USA). Japanese users can download Flu-Report free of charge from the iPhone App Store. Flu-Report collects information about influenza virus infections based on a self-administered questionnaire. The main survey items were sex, age, home postal code, and influenza infection status. Screenshots of the survey report are shown in Multimedia Appendices 1 and 2. Information was also collected on any relatives who lived with participants, including the age and influenza infection status of each relative. In addition, when a participant or their relative developed physician-diagnosed influenza, information about the type of influenza and the names of prescribed antivirals were collected. In Flu-Report, influenza infections are defined as influenza diagnosed by a physician or antiviral medication prescribed by a physician. Self-reported influenza virus infections that did not meet these criteria were defined as influenza-like illnesses (ILI).

### Study Population

This prospective observational study evaluated the use of Flu-Report to investigate influenza virus infections in Japan from November 2016 to March 2017. All iPhone users in Japan aged ≥20 years who agreed to join the study were able to participate in our survey. To recruit volunteers, we advertised Flu-Report on TV programs, posted articles about Flu-Report on the internet, and displayed posters about Flu-Report at cooperating medical facilities.

### Data Collection

Participants who agreed to participate in the survey were encouraged to complete the influenza questionnaire on their iPhone. All participants were instructed to enter demographic information (age, sex, and home postal code). Subsequently, if a participant or their registered family members developed an influenza infection, participants entered additional information into the questionnaire, namely: onset date, name of prescribed antiviral medicines, and symptoms. Participants were expected to report as soon as possible when they or their relatives developed an influenza infection. However, participants could report that they had developed an influenza infection at a later date. Flu-Report also issued a message on the iPhone screen each month to remind participants to report any influenza infection. Participants could select the brand name of any prescribed antivirals from a list provided: oseltamivir phosphate (Tamiflu), zanamivir hydrate (Relenza), laninamivir octanoate hydrate (Inavir Dry Powder Inhaler), peramivir hydrate (RAPIACTA), others, and unknown. Participants were also asked to select symptoms of influenza infection from a displayed list: fever, cough, muscle pain, malaise, headache, sore throat, sneezing, runny nose, chills (enough to wear a coat), chills (requiring a thick blanket), chills (cannot stop trembling), and all symptoms. Screenshots of the influenza questionnaire are shown in Multimedia Appendix 2.

**Figure 1:**
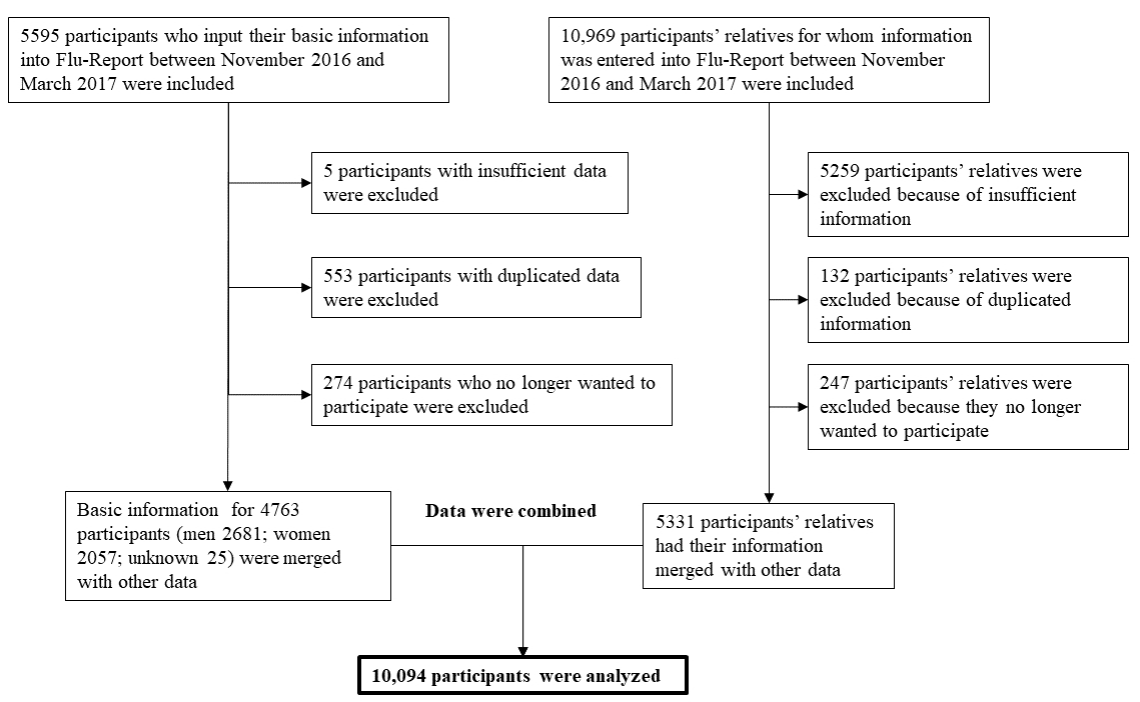
Inclusion and exclusion criteria for subjects who input basic information into Flu-Report.

**Figure 2:**
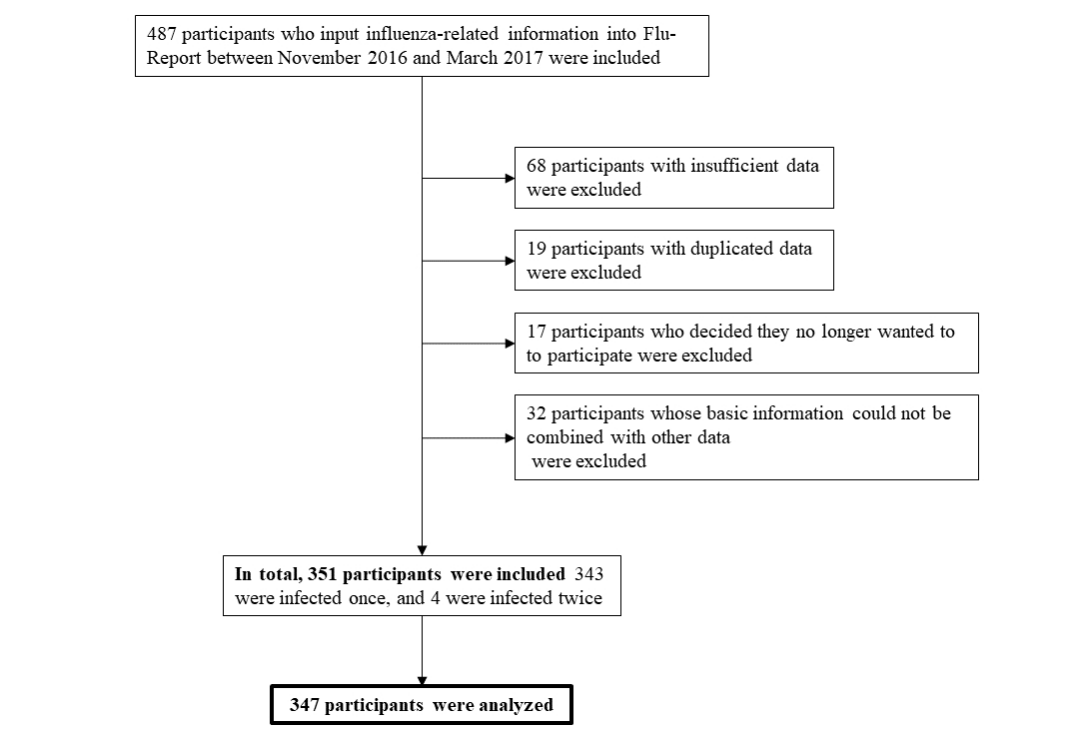
Inclusion and exclusion criteria for subjects who input influenza-related information into Flu-Report.

This study analyzed participants’ background information and influenza virus infection status data. Inclusion and exclusion criteria are shown in Figures 1 and 2. Figure 1 shows the number of people who agreed to participate, the number of participants excluded, and the number of participants included in the final analysis. Initially, information for 5595 participants and 10,969 cohabitating relatives was entered in Flu-Report.

Reasons for exclusion were insufficient information, duplicated information, and withdrawal of consent. Finally, 4763 participants (2681 men; 2057 women; unknown 25) and 5331 cohabiting relatives were included, giving a total of 10,094 participants for the analysis.

Figure 2 shows the number of participants with influenza or ILI who registered, the number of participants who were excluded, and the number of participants finally analyzed in this study. Of these, 334 participants were considered to have been infected with influenza virus, and 13 were classified as ILI.

### Data Handling

First, information about seasonal influenza activity obtained via Flu-Report was compared with basic information from a real-time prescription surveillance system [7,8]. Japan recently launched a real-time prescription surveillance system (ie, an automatic surveillance system based on electronic records regarding prescription drug purchases). This system automatically collects information about prescriptions of antiviral medication from the electronic prescription record system (including information from over 10,000 pharmacies) and estimates the number of influenza cases based on this information. This system has been shown to be able to detect influenza activity earlier than the conventional influenza survey systems, and the results displayed a good correlation with data for influenza epidemics obtained using the conventional influenza survey systems [7]. In Japan, influenza activity is shown as the number of individuals with influenza during a specific period of time. To evaluate trends in influenza activity, the correlation between Flu-Report-derived influenza infection time series data and basic information obtained from the real-time prescription surveillance system was estimated. In addition, trends for morbidity and influenza activity were evaluated using data for participants, data for cohabiting relatives, and combined data for participants and cohabiting relatives. We analyzed data using the “date diagnosed with influenza” that participants entered into Flu-Report. Second, the research period was divided into 3 periods (first to third periods) every 4 weeks, and 2 periods every 5 weeks (fourth and fifth periods) according to the calendar. We have shown the distribution of subjects with influenza or ILI in Flu-Report and the real-time prescription surveillance system for each period. The distribution was calculated by dividing the number of participants with influenza or ILI in each period by the total number of participants with influenza or ILI for all periods. Third, the accuracy of the self-reported information about influenza and ILI provided via Flu-Report was evaluated through comparison with recent estimates of the number of influenza patients in Japan. The estimated number of patients with influenza in Japan was calculated based on reports from the Japanese Ministry of Internal Affairs and Communications and the Ministry of Health, Labour and Welfare [9,10]. In our study, we defined influenza cases per observed persons as the “morbidity ratio.”

### Statistical Analysis

The correlation between the 2 collection systems was estimated using Spearman rank correlation coefficient. All calculations were performed using the statistical software JMP Pro version 11 (SAS Institute Inc, Cary, NC, USA). *P*<.05 was considered statistically significant.

## Results

### Trends in Influenza Activity

**Figure 3:**
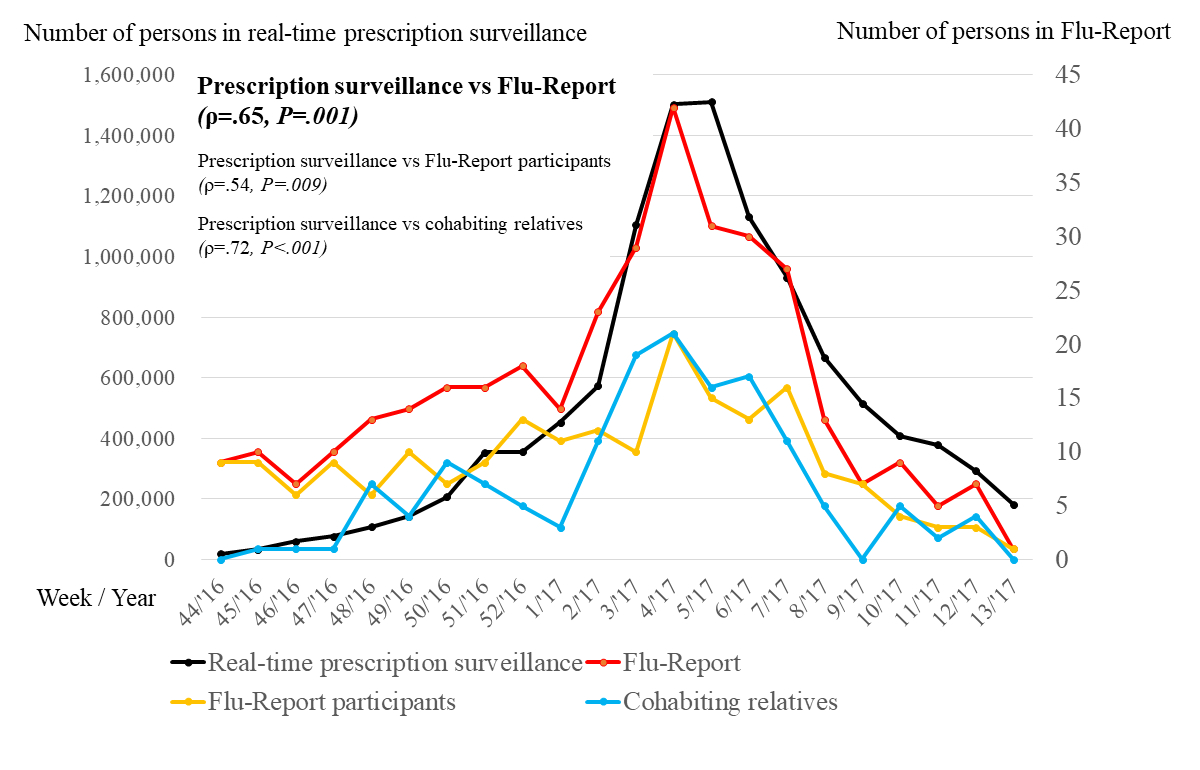
The correlation between Flu-Report data and basic information for the real-time prescription surveillance system.

Figure 3 shows the correlation between Flu-Report data and basic information from the real-time prescription surveillance system regarding trends in influenza activity. Flu-Report-derived influenza and ILI infection time series data (the combined data for participants and cohabiting relatives) were significantly associated with basic data for the real-time prescription surveillance system (rho, ρ=.65, *P*=.001). In addition, a similar tendency was found when using data for participants and data for cohabiting relatives (ρ=.54, *P*=.009; ρ=.72, *P*<.001, respectively; see Multimedia Appendix 3 for raw data).

In total, 64.6% (224/347) of influenza-infected participants registered their information on the day of their diagnosis or the next day, and 79.3% (275/347) registered their information within 3 days. Approximately 92.8% (322/347) of participants registered their information within 7 days (data not shown).

**Table 1:** Influenza morbidity ratios for study participants based on Flu-Report. All data were drawn from Flu-Report as entered by participants in this study.

Age, years		Participants, N	Participants affected by influenza, n (%)	Participants affected by ILI^a^, n (%)
**<20**		2134	108 (5.06)	4 (0.19)
	<1	107	1 (0.9)	0 (0.0)
	1-5	522	27 (5.2)	1 (0.2)
	6-12	808	46 (5.7)	1 (0.1)
	13-19	697	34 (4.9)	2 (0.3)
**20-59**		6958	220 (3.16)	9 (0.13)
	20-29	1705	78 (4.57)	5 (0.29)
	30-39	1694	61 (3.60)	1 (0.06)
	40-49	2103	60 (2.85)	3 (0.14)
	50-59	1456	21 (1.44)	0 (0.00)
≥**60**		1002	6 (0.60)	0 (0.00)
	60-69	621	2 (0.3)	0 (0.0)
	70-79	233	3 (1.3)	0 (0.0)
	≥80	148	1 (0.7)	0 (0.0)
Total		10,094	334 (3.31)	13 (0.13)

^a^ILI: influenza-like illness.

**Table 2:** Estimated influenza morbidity ratios for the general Japanese population according to age, based on Japanese Government reports. All data were drawn from reports of the Japanese Ministry of Internal Affairs and Communications and the Ministry of Health, Labour and Welfare.

Age, years	Population of Japan in 2016^a^, N	Estimated number of patients with influenza during 2013-2014^a^, n (%)	Estimated number of patients with influenza during 2014-2015^a^, n (%)	Estimated number of patients with influenza during 2015-2016^a^, n (%)
<20	21.74	8.24 (37.90)	6.95 (31.97)	7.95 (36.57)
20-59	62.16	5.07 (8.16)	5.64 (9.07)	5.40 (8.69)
≥60	42.85	1.16 (2.71)	1.88 (4.39)	1.65 (3.85)
Total	126.76	14.46 (11.41)	14.47 (11.42)	15.02 (11.85)

^a^Per million people.

Multimedia Appendix 4 shows the distributions of participants with influenza or ILI during each period. The distribution of participants in Flu-Report appeared to be greater than those in the real-time prescription surveillance system in the first and second periods and less in the fourth and fifth periods (see Multimedia Appendix 3 for raw data).

### Influenza Morbidity Ratios

Table 1 shows the influenza morbidity ratios for our participants. Table 2 shows morbidity ratios for the Japanese population.

The age distribution of our participants was as follows: 2134 (21%) aged <20 years, 6958 (69%) aged 20-59 years, and 1002 (10%) aged ≥60 years. The estimated number of patients with influenza in Japan was calculated based on reports by the Japanese Ministry of Internal Affairs and Communications and the Ministry of Health, Labour and Welfare [9,10]. The influenza morbidity ratio from Flu-Report was 5.1% among those aged <20 years, 3.2% among those aged 20-59 years, and 0.6% among those aged ≥60 years. In contrast, the influenza morbidity ratios for Japanese individuals aged <20 years, 20-59 years, and ≥60 years were 32.0%-37.4%, 8.2%-9.1%, and 2.7%-4.4%, respectively.

## Discussion

### Principal Findings

This study investigated the utility of Flu-Report, a new flu-tracking iPhone app, to monitor influenza activity. Flu-Report gathered information for over 10,000 participants. We found that Flu-Report-derived influenza infection time series data showed a good fit with equivalent information obtained by an existing large-scale real-time prescription surveillance system. Therefore, Flu-Report may be an effective tool for real-time detection of influenza cases. To our knowledge, this is the first report about a new influenza-tracking system based on an iPhone app developed using “ResearchKit” in Japan.

Surveillance of influenza activity is generally labor-intensive. For influenza surveillance, the annual influenza activity survey in Japan is conducted with the cooperation of about 5000 medical institutions. The advantage of Flu-Report is that a large amount of information can be obtained through a small amount of capital and labor. A previous study revealed that a mobile phone–based data collection system delivered data faster, produced fewer errors, and had lower running costs than a paper-based data collection system [11]. Therefore, surveillance of influenza activity via a mobile phone app is a feasible option for research under financial constraints. Incidentally, in our study, influenza morbidity ratios for individuals aged <20 years, 20-59 years, and ≥65 years obtained using Flu-Report were lower than influenza morbidity rates among equivalent sections of the Japanese population (approximately one-seventh, one-third, and one-seventh, for the 3 age groups, respectively). Regarding flu morbidity, the older age group had a lower morbidity ratio in both Flu-Report and surveillance information, suggesting that Flu-Report collected influenza development by age category to some extent. However, our results also suggest that Flu-Report has some limitations associated with influenza surveillance using information and communications technologies. A previous report showed that nowcasting and forecasting ILI using Web queries have different correlations in different age categories [12]. That report will help in interpreting our results in that use of information and communications technologies on personal devices vary by age categories (ie, adults frequently use a mobile phone, whereas older adults do not). This means that the user population of Flu-Report is affected by selection bias, that is, bias that occurs when selecting individuals or groups to participate in the study. Moreover, we speculate that study participants were more concerned about the flu and more frequently used information and communications technologies compared with the general Japanese population. Therefore, participants’ age and health-conscious behaviors must be considered when evaluating disease surveillance using information and communications technology devices.

As mentioned, there are various strengths and limitations to influenza surveillance systems using information and communications technologies. Previous reports have shown that a combination of multiple datasets improves the ability of models designed to predict influenza outbreaks [13-15]. Combining information obtained using Flu-Report with data collected using other influenza surveillance systems may facilitate effective influenza outbreak surveillance.

### Limitations

Several other limitations of this study need to be acknowledged. First, this study only obtained influenza-related information for one season. It is known that the epidemic strains of influenza change each year. Therefore, the extent of the spread of influenza infections differs each season. Further evaluations must include additional influenza seasons. Second, our study contained an insufficient number of participants to allow us to investigate influenza activity that occurs throughout Japan. More participants are needed to enhance the effectiveness of Flu-Report. Another limitation was that there were fewer influenza infection reports via Flu-Report than expected. In Flu-Report, many influenza infections were reported earlier in the influenza season, and influenza infection reports decreased later in the season. There were some cases in which our reminder system for influenza infection each month did not work well. This error may have caused a reduction of infection reports during the latter part of the influenza season. Finally, all data were obtained using a self-administered questionnaire, and participants might have provided inaccurate information. Unfortunately, it appears to be difficult to confirm data accuracy or response rates of self-administered survey questionnaires using mobile apps [16]. This is an issue to be resolved in future surveillance using information and communications technologies.

### Comparison With Prior Work

Various influenza monitoring systems based on the internet, search engine query data, or Twitter data have recently been launched [4,5]. These systems can estimate influenza activity faster than the conventional influenza surveillance systems. However, it has been reported that there are discrepancies between information obtained via the internet, search engine query data, or Twitter data, and data collected via the conventional systems [17]. Extensive media reports about influenza may promote influenza-related searches by people without influenza, which could have affected the results obtained using internet-based systems. Flu-Report, which focuses on only collecting influenza-related information, may be less influenced by such issues.

Similar influenza participatory surveillance systems are already in operation around the world [18-20]. These systems allow users to report the presence or absence of ILI symptoms each week, and ask follow-up questions about health care seeking behavior and diagnosed influenza. However, our system allows participants to first report information on influenza diagnosis by a physician and/or antiviral prescriptions. In addition, our system encourages participants to report on the presence or absence of influenza infection each month. As an influenza epidemic survey method, the former systems emphasize sensitivity, whereas the latter system increases specificity. In the case of a survey system targeting seasonal influenza (which is strongly infectious and prevalent on a large scale in a short period of time), the former system types are thought to be more suitable. Flu-Report may be better suited for monitoring diseases that are not so high in terms of infectivity but are serious when infected.

### Conclusions

Although information obtained via Flu-Report is affected by selection bias, Flu-Report makes it easy to obtain near real-time information about influenza activity via the accumulation of self-administered questionnaires using mobile phone. Flu-Report has potential to provide basic data that could help to detect influenza outbreaks.

## Appendix

Multimedia Appendix 1 Screenshots for format of survey.

Multimedia Appendix 2 Screenshots for the influenza questionnaire.

Multimedia Appendix 3 The raw data of the correlation between Flu-Report data and basic information for the real-time prescription surveillance system.

Multimedia Appendix 4 The distribution of subjects with influenza or influenza-like illness during each period.

## Abbreviations

ILI: influenza-like illnesses
