# Supplementary material for: A New Influenza-Tracking Smartphone App (Flu-Report) Based on a Self-Administered Questionnaire: Cross-Sectional Study
Source: JMIR Mhealth Uhealth. 2018 Jun 6;6(6):e136. doi: 10.2196/mhealth.9834 (PMC6010834; doi:10.2196/mhealth.9834)
Supplement: Multimedia Appendix 1 [file mhealth_v6i6e136_app1.pptx]

## Slide 1
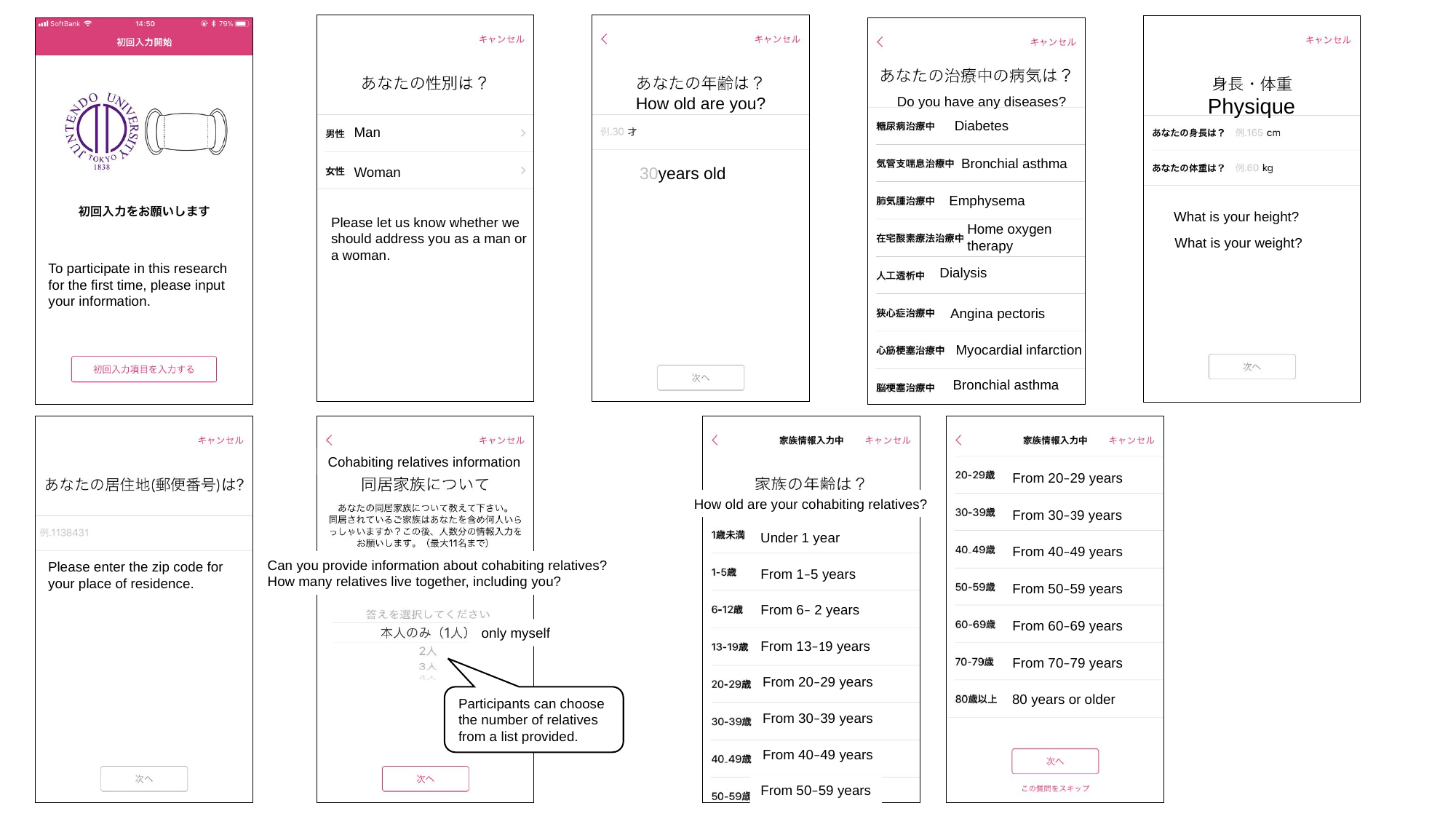

How old are you?
 Do you have any diseases?
Physique
 Diabetes
Man
Bronchial asthma
30years old
Woman
Emphysema
What is your height?
Please let us know whether we should address you as a man or a woman.
Home oxygen
therapy
What is your weight?
To participate in this research for the first time, please input your information.
Dialysis
Angina pectoris
Myocardial infarction
Bronchial asthma
Cohabiting relatives information
From 20–29 years
How old are your cohabiting relatives?
From 30–39 years
Under 1 year
From 40–49 years
Can you provide information about cohabiting relatives?
How many relatives live together, including you?
Please enter the zip code for
your place of residence.
From 1–5 years
From 50–59 years
From 6– 2 years
From 60–69 years
only myself
From 13–19 years
From 70–79 years
From 20–29 years
80 years or older
Participants can choose the number of relatives from a list provided.
From 30–39 years
From 40–49 years
From 50–59 years
