# Supplementary material for: A New Influenza-Tracking Smartphone App (Flu-Report) Based on a Self-Administered Questionnaire: Cross-Sectional Study
Source: JMIR Mhealth Uhealth. 2018 Jun 6;6(6):e136. doi: 10.2196/mhealth.9834 (PMC6010834; doi:10.2196/mhealth.9834)
Supplement: Multimedia Appendix 2 [file mhealth_v6i6e136_app2.pptx]

## Slide 1
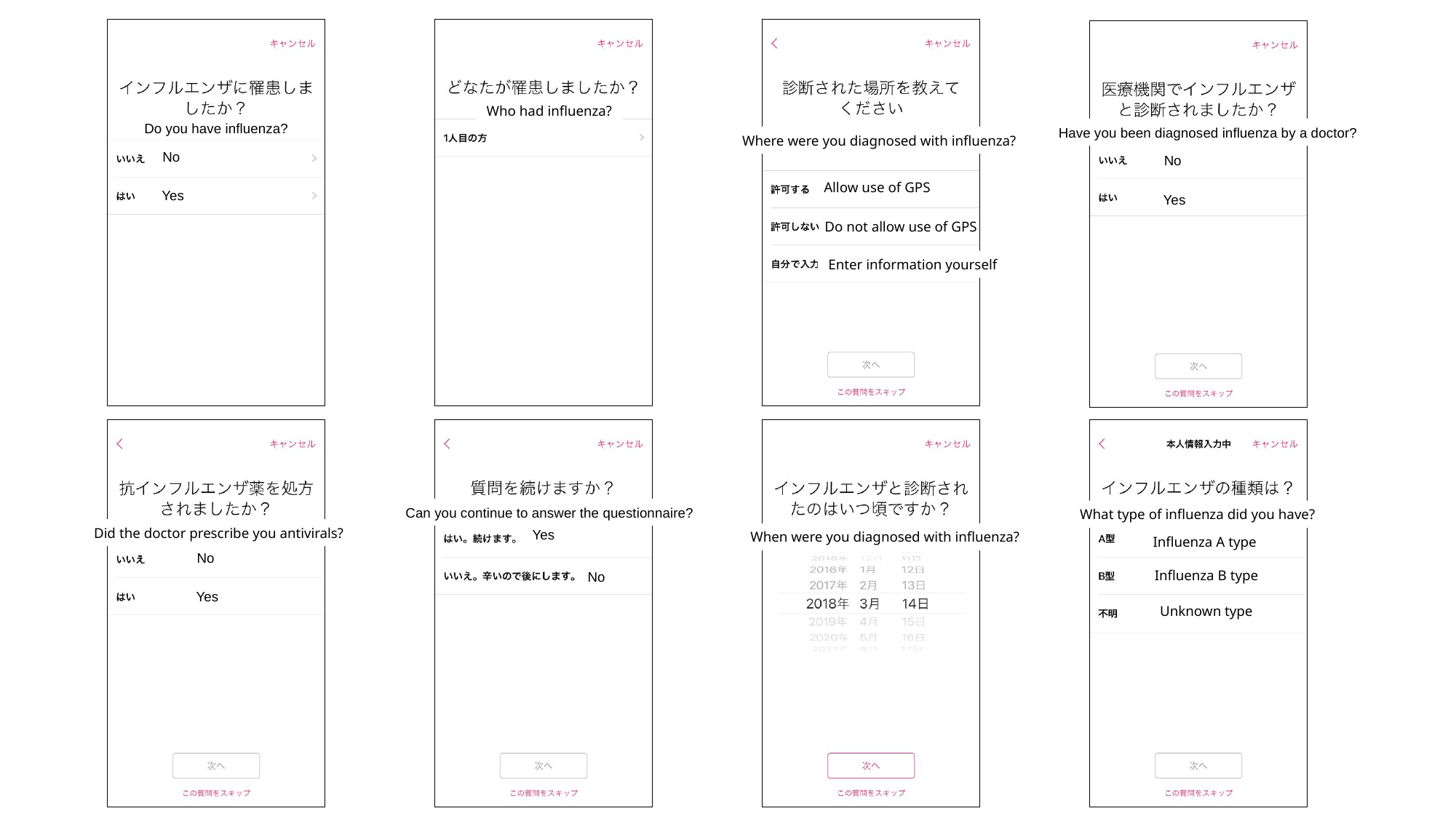

Who had influenza?
Do you have influenza?
Have you been diagnosed influenza by a doctor?
Where were you diagnosed with influenza?
No
No
Allow use of GPS
Yes
Yes
Do not allow use of GPS
Enter information yourself
Can you continue to answer the questionnaire?
What type of influenza did you have?
Did the doctor prescribe you antivirals?
Yes
When were you diagnosed with influenza?
Influenza A type
No
Influenza B type
No
Yes
Unknown type

## Slide 2
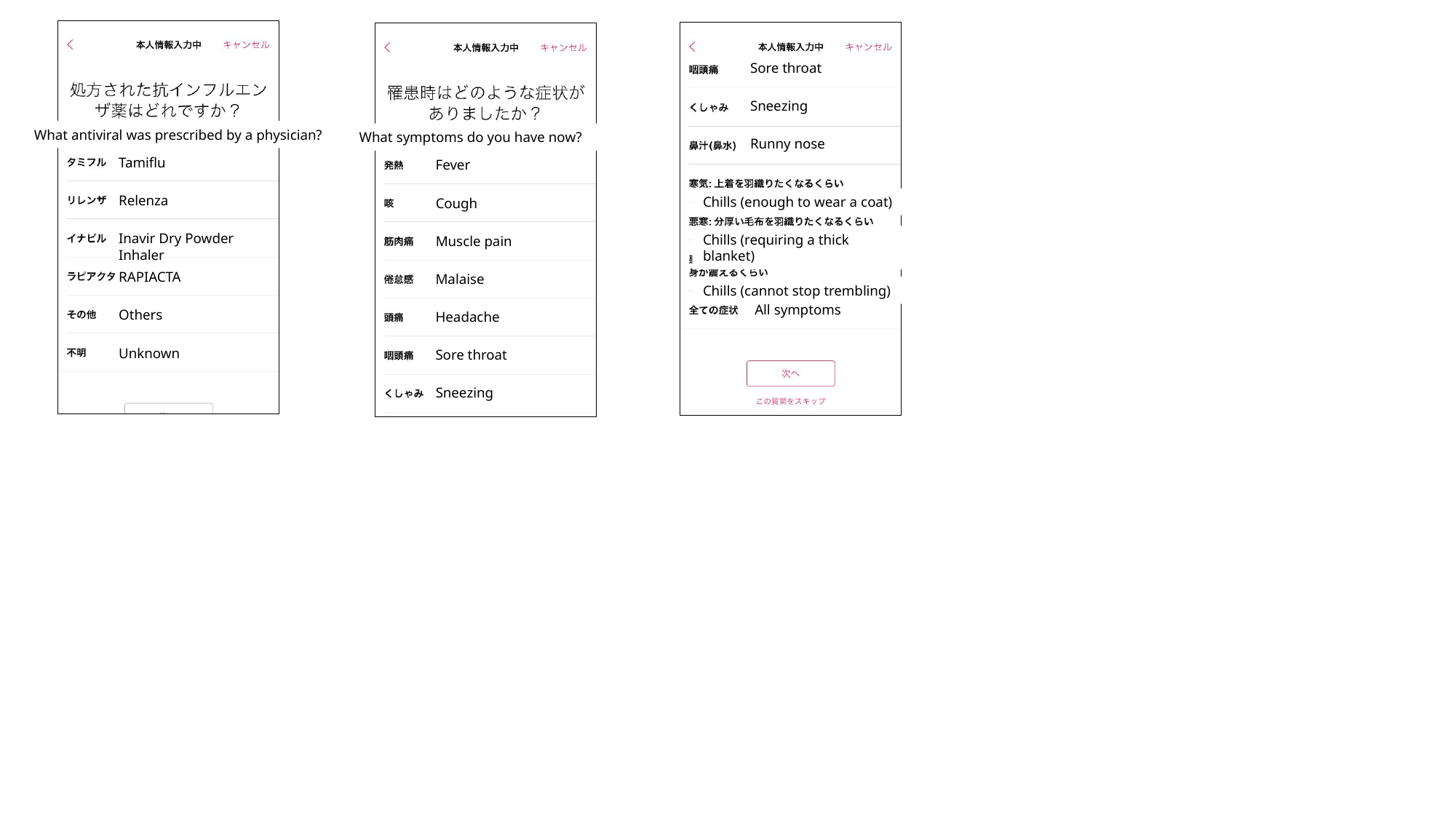

Sore throat
Sneezing
What antiviral was prescribed by a physician?
What symptoms do you have now?
Runny nose
Tamiflu
Fever
Relenza
Chills (enough to wear a coat)
Cough
Inavir Dry Powder Inhaler
Chills (requiring a thick blanket)
Muscle pain
RAPIACTA
Malaise
Chills (cannot stop trembling)
All symptoms
Others
Headache
Unknown
Sore throat
Sneezing
