# Supplementary material for: A New Influenza-Tracking Smartphone App (Flu-Report) Based on a Self-Administered Questionnaire: Cross-Sectional Study
Source: JMIR Mhealth Uhealth. 2018 Jun 6;6(6):e136. doi: 10.2196/mhealth.9834 (PMC6010834; doi:10.2196/mhealth.9834)
Supplement: Multimedia Appendix 4 [file mhealth_v6i6e136_app4.pptx]

## Slide 1
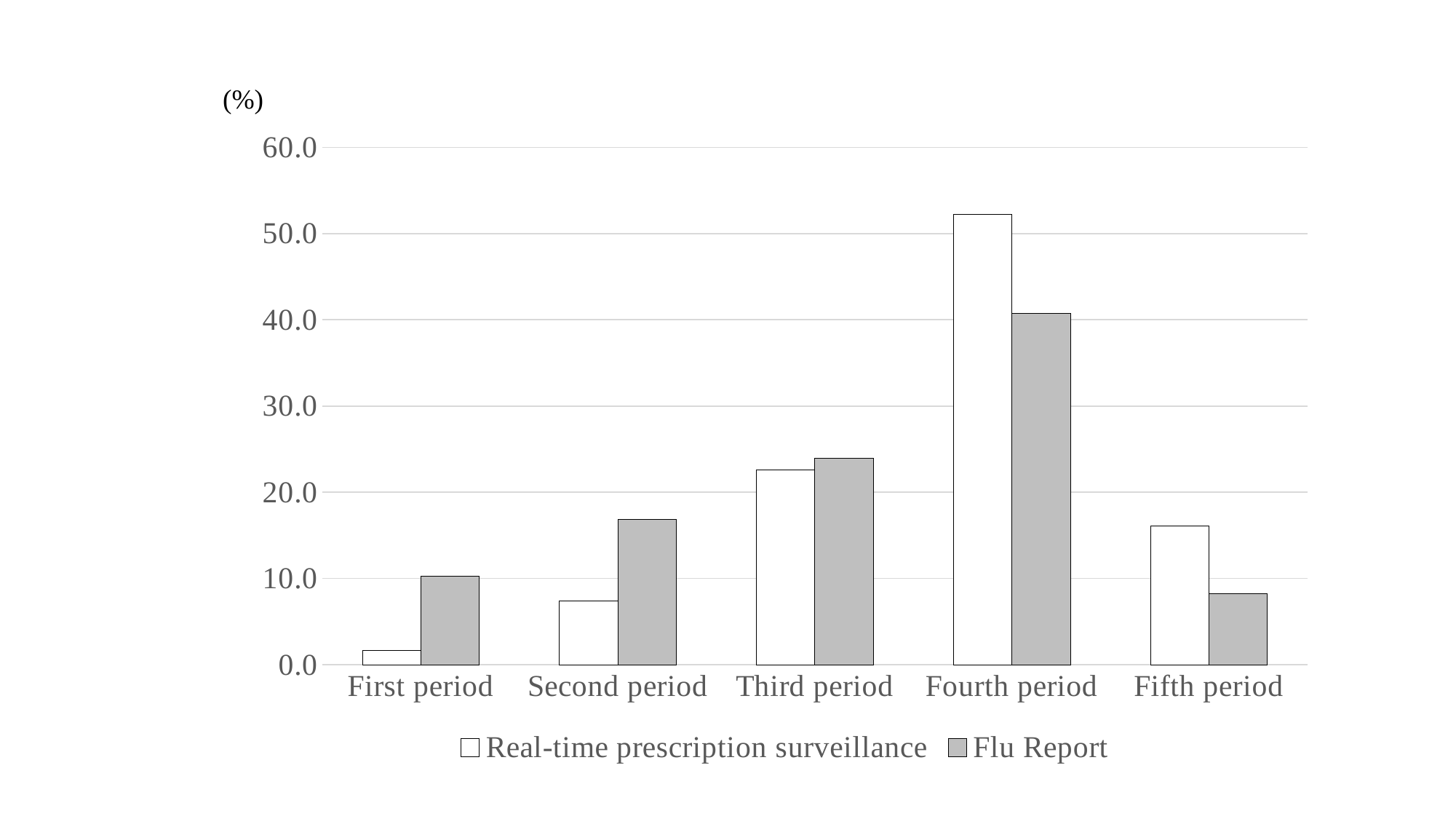

(%)
### Chart
| Category | Real-time prescription surveillance | Flu Report |
|---|---|---|
| First period | 1.693860765884796 | 10.256410256410255 |
| Second period | 7.381221742591007 | 16.80911680911681 |
| Third period | 22.592548056057502 | 23.931623931623932 |
| Fourth period | 52.20893488331383 | 40.74074074074074 |
| Fifth period | 16.123434552152872 | 8.262108262108262 |
